# Telomerase reverse transcriptase promoter mutations in hepatitis B virus-associated hepatocellular carcinoma

**DOI:** 10.18632/oncotarget.8539

**Published:** 2016-04-01

**Authors:** Xunjun Yang, Xiuchan Guo, Yao Chen, Guorong Chen, Yin Ma, Kate Huang, Yuning Zhang, Qiongya Zhao, Cheryl A. Winkler, Ping An, Jianxin Lyu

**Affiliations:** ^1^ Key Laboratory of Laboratory Medicine, Ministry of Education, Zhejiang Provincial Key Laboratory of Medical Genetics, Wenzhou Medical University, Wenzhou, Zhejiang, China; ^2^ Department of Laboratory Medicine, The Second Affiliated Hospital & Yuying Children's Hospital of Wenzhou Medical University, Wenzhou, Zhejiang, China; ^3^ ICF International, Atlanta, GA, USA; ^4^ Department of Pathology, The First Affiliated Hospital of Wenzhou Medical University, Wenzhou, Zhejiang, China; ^5^ Basic Research Laboratory, Center for Cancer Research, National Cancer Institute, Leidos Biomedical Research, Inc., Frederick National Laboratory for Cancer Research, Frederick, MD, USA

**Keywords:** hepatocellular carcinoma, TERT, mutation, telomerase reverse transcriptase

## Abstract

Telomerase reverse transcriptase (*TERT*) promoter mutations are among the most frequent noncoding somatic mutations in multiple cancers, including hepatocellular carcinoma (HCC). The clinical and pathological implications of *TERT* promoter mutations in hepatitis B virus (HBV)-associated HCC have not been resolved. To investigate *TERT* promoter mutations, protein expression, and their clinical-pathological implications, we sequenced the *TERT* promoter region for hotspot mutations in HCC tissues and performed immunostaining for TERT protein expression from HBV-associated HCC in Chinese patients. Of 276 HCC tumor DNA samples sequenced, 85 (31%) carried *TERT* promoter mutations. *TERT* promoter mutations were more frequent in those with low α-fetoprotein (AFP) serum levels (*p* = 0.03), advanced age (*p* = 0.04), and in those lacking HCC family history (*p* = 0.02), but were not correlated with HCC stages and grades. TERT protein levels were higher in HCC (n = 28) compared to normal liver tissues (n = 8) (*p* =0.001), but did not differ between mutated and non-mutated tumor tissues. In conclusion, *TERT* promoter mutations are common somatic mutations in HCC of Han Chinese with HBV infection. Detection of *TERT* promoter mutations in those with low levels of AFP may aid diagnosis of HCC with atypical presentation.

## INTRODUCTION

Hepatocellular carcinoma (HCC) is the fifth most common cancer worldwide and the third leading cause of cancer-related deaths in China [1, 2]. Patient survival relies on early diagnosis, which remains challenging due to the lack of sensitive and specific tumor markers [3, 4]. HCC develops typically in the setting of cirrhosis associated with hepatitis B virus (HBV) infection in China [1, 4, 5]. HCC carcinogenesis involves multiple genetic changes including loss of tumor suppressor genes and activation of oncogenes, while HBV infection caused liver inflammation may induce mutation accumulation in hepatocytes [6]. The precise molecular mechanism of HBV-associated HCC remains unresolved.

Telomerase synthesizes telomeric TTAGGG repeats essential for chromosome stability and sustained cellular proliferation [7]. In normal somatic cells, length of telomeres shortens at each cycle of cell division, directing cells towards senescence and apoptosis. Telomerase is silent in most normal differentiated cells, while it is activated in up to 90% of human malignancies including HCC, leading to infinite proliferation potential [8–10]. The cancer-specific telomerase activation is primarily determined by telomerase reverse transcriptase (TERT) activity, which may be re-activated by epigenetic regulation, *TERT* amplification or *TERT* promoter mutations [8, 11, 12].

*TERT* promoter mutations have been found in many human cancers, including melanomas [12, 13], bladder carcinomas [14, 15], and thyroid carcinomas [16–18]. The mutations mainly occur in two hotspot sites located -124 (C228T) and -146 bp (C250T) upstream from the ATG start site, both of which create binding motifs for Ets/TCF transcription factors [19]. Either *TERT* promoter mutation is reported to elevate gene transcriptional activity by 2-4 fold [12].

*TERT* promoter mutations have recently been identified in 59% of HCC tissues from European patients with various etiologies [20]. *TERT* promoter mutations were also found in premalignant lesions within fibrous tissues, and thus are thought to be a new biomarker predictive of transformation of premalignant lesions into HCC [21]. Previous studies for *TERT* promoter mutations in HCC have been performed in in individuals infected with HCV from western countries and in Japan [11, 22–25]. However, little is known in patients with HCC secondary to HBV infection in Han Chinese, a population with the highest mortality rates. In the present study, we have determined promoter mutation status, TERT protein expression, and their clinical-pathological implications in HBV-associated HCC in 276 Chinese patients with comprehensive clinical, viral, and pathological data.

## RESULTS

### *TERT* promoter hotspot mutations in HCC

**Figure 1:**
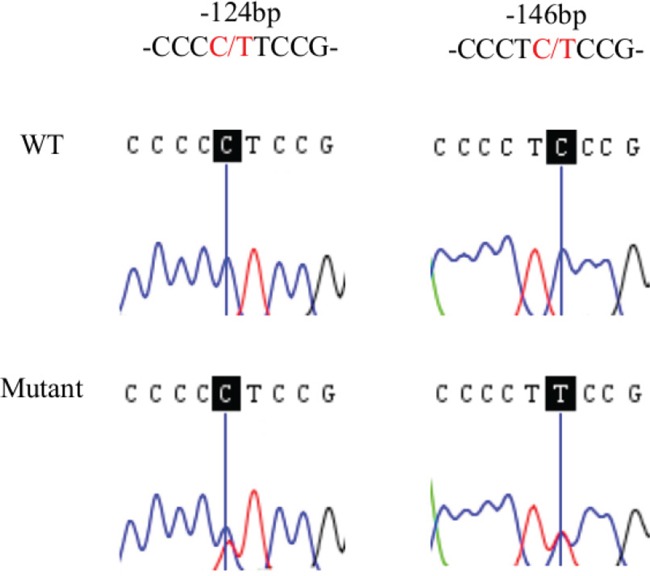
Sequence analysis of *TERT* promoter somatic mutations in HCC Sanger sequencing of mutant or wildtype (WT) of the *TERT* promoter mutations at positions -124 and -146 bp (shadowed) from representative cases.

By direct sequencing of the *TERT* promoter region, we detected *TERT* hot spot mutations in 85 (31%) tumor tissues from 276 HCC cases (Table 1, Supplementary Figure S1). C228T was detected in 84 HCC tumors (30%) and C250T was detected in 1 HCC tumor (0.4%) (Figure 1). The two mutations were mutually exclusive, consistent with published observations [19]. In contrast, we found no *TERT* mutation in liver tissues from 20 control patients with cirrhosis or hepatolithiasis without HCC. *TERT* promoter mutations were significantly higher in HCC than in non-HCC patients (*p* = 0.003, Fisher Exact test).

**Table 1:** Characteristics of the HCC patients according to *TERT* promoter mutation status

Characteristics	HCC, Total (N=276)	*TERT* promoter mutation status		OR	95%CI	*p* value^a^
		Mutated N=85 (%)	Non-Mutated N = 191 (%)			
Number of tumor		85 (30.8)	191 (69.2)			
Gender						
Female	41	10(24.4)	31(75.6)	1.00	Reference	
Male	235	75(31.9)	160(68.1)	1.45	0.68-3.12	0.34
Age median	276	57.8±9.6	56.1±11.8			0.21^b^
< 60 yr.	158	41(25.9)	117(74.1)	1.00	Reference	
≥60 yr.	118	44(36.8)	74(63.2)	1.70	1.01-2.84	0.04*
HBV marker (HBsAg)						
Negative	60	17(28.3)	43(71.7)	1.00	Reference	
Positive	215	68(31.6)	147(68.4)	1.17	0.62-2.20	0.63
HBV marker (HBcAb or HBe Ab +)						
Negative	16	7 (43.8)	9 (56.2)	1.0		
Positive	259	78 (30.1)	181 (69.9)	0.55	0.20-1.54	0.27
Family history						
No	238	81(34.0)	157(66.0)	1.00	Reference	
Yes	26	3(11.5)	23(88.5)	0.25	0.07-0.87	0.02*
Tumor size						
<3cm	78	23(29.5)	55(70.5)	1.00	Reference	
3-5cm	82	29(35.4)	53(64.6)	1.31	0.67-2.54	0.43
>5cm	53	13(24.5)	40(75.5)	0.77	0.35-1.72	0.53
Cirrhosis						
No	52	22(42.3)	30(57.7)	1.00	Reference	
Yes	195	62(31.8)	133(68.2)	0.64	0.34-1.19	0.16
Tumor Embolus						
No	109	32(29.4)	77(70.6)	1.00	Reference	
Yes	41	13(31.7)	28(68.3)	1.11	0.51-2.43	0.78
Tumor Capsule						
No	20	6(30)	14(70)	1.00	Reference	
Yes	163	43(26.4)	120(73.6)	0.84	0.30-2.31	0.73
Clinical stages						
I	40	12(30)	28(70)	1.00	Reference	
II	169	56(33.1)	113(66.9)	1.16	0.55-2.44	0.70
III	40	9(22.5)	31(77.5)	0.68	0.25-1.85	0.45
IV	17	4(23.5)	13(76.5)	0.72	0.19-2.66	0.86
Tumor differentiation						
I	35	9(25.7)	26(74.3)	1.00	Reference	
II	159	51(32.1)	108(67.9)	1.36	0.60-3.12	0.46
III	67	21(31.3)	46(68.7)	1.31	0.53-3.30	0.55

a *p* values were from *χ2* test, except for

b which was from t test

* *p* < 0.05.

### Clinical and pathological features of *TERT* promoter mutations in HCC Patients

Clinicopathological data, including demographic characteristics, laboratory results for HBV infection and liver function, and histopathology findings, were used for analysis. The relationship between *TERT* promoter mutation status (mutated/non-mutated) and clinicopathological features in HCC patients are presented in Table 1.

The presence of the *TERT* mutation was not associated with sex, HBV infection status, or the presence of HBV DNA, cirrhosis, tumor embolus, tumor capsule or tumor size (Table 1). *TERT* mutations were also not associated with HCC stages and tumor differentiation grades (Table 1) or with liver function tests (Supplementary Table S1). However, we found that HCC patients with a first-degree relative with HCC were less likely to carry *TERT* mutations (12%) compared to those without a family history (34%) (*p* = 0.02). HCC patients ≥60 yr were more likely to carry *TERT* mutations (37%) compared to those <60 yr (26%) (*p* = 0.04) (Table 1).

### Correlation of *TERT* promoter mutations and α-fetoprotein (AFP) levels in HCC patients

**Figure 2:**
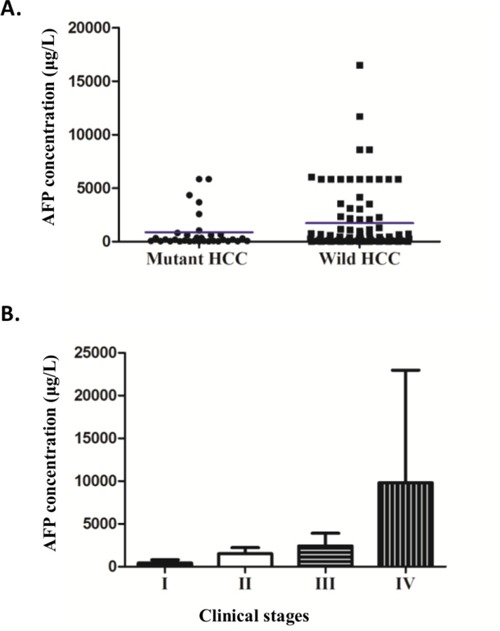
AFP levels in relationship with *TERT* promoter mutations and clinical stages **A.** The scatter plot of the concentration of serum AFP (ug/L) in *TERT* mutant and wild-type groups (*p* = 0.045 excluded 2 outlier data points of more than 54000 in wild-type group; *p* = 0.03 including all data points as show in Supplementary Figure S2). Mean is shown as blue horizontal line. **B.** The relationship of AFP (ug/L) with clinical stages, ANOVA analysis (AFP>20, *p*=0.002).

*TERT* mutant carriers had lower serum AFP levels than the non-mutated group. The cutoff of AFP > 200 μg/l is standard in clinical practice in China to monitor HCC development in HBV infected patients [26, 27]. Mutation carriers (18.2%) were less likely to have AFP > 200 μg/l compared to the non-mutated group (18.2% versus 30.8%, respectively; *p* = 0.04, OR 0.5, 95% CI = 0.26-0.97, Table 2). Moreover, a sensitivity analysis of all HCC cases with abnormal AFP levels (using the conventional abnormal cutoff at AFP > 20μg/l) indicated *TERT* promoter mutations were significantly associated with lower AFP levels (*p* = 0.03; Figure 2A, Supplementary Figure S2, Table 2). Since HCC is more prevalent in males, we performed a stratified analysis for males and females; *TERT* promoter mutations were significantly associated with AFP levels and HBsAg positive males (*p* = 0.04), with adjustment for age.

**Table 2:** The AFP levels in HCC patients according to the *TERT* promoter mutation status

	AFP Group	Mutated (n = 77)	Non-Mutated (n = 172)	*p*-value	OR	95% CI
➀	AFP ≤ 20	44 (57.1%)	83 (48.3%)	0.20^a^	0.70	0.41-1.20
➁	20 < AFP ≤ 200	19 (24.7%)	36 (20.9%)			
➂	AFP > 200	14 (18.2%)	53 (30.8%)	0.038^b^	0.50	0.26-0.97
				0.09^c^	0.50	0.22-1.12
	AFP quantity mean±SD (> 20)	870 ± 1638.7	1732.8 ± 2927.7	0.03*		

Note:

a ➀vs➁+➂

b ➀+➁vs➂

c ➁vs➂, from *χ2* test

* from *t* test.

We further observed a strong positive correlation of AFP levels and clinical stages (ANOVA AFP>20, *p* =0.002, Figure 2B), with AFP increasing with advancing stages. This result is consistent with previous findings in Chinese populations [28, 29]. Together, these data suggest that in those individuals with low AFP levels, detection of *TERT* promoter mutations may provide additional evidence for HCC diagnosis.

### TERT protein expression measured by Immunohistochemistry (IHC)

**Figure 3:**
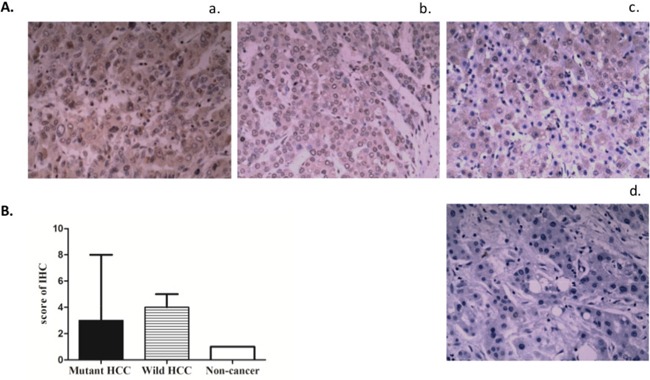
Expression of TERT protein in liver tissues stratified by *TERT* promoter mutation status **A.** Immunostaining of TERT in representative cases. a: *TERT* promoter mutated live cancer; b: *TERT* promoter non-mutated live cancer; c: Non-cancer liver tissue (cirrhosis or hepatolith); d: negative control (without antibody to hTERT). Note the TERT staining in the nucleus. **B.** Bar graph of IHC scores representing TERT protein levels (Median with interquartile rage); mutant (n = 15) vs wild type (n = 13): *p* =0.94; HCC (n = 28) vs non-cancer (n= 8): *p* =0.0013 (U test).

We measured TERT protein expression by IHC in 28 HCCs (15 with mutation and 13 without mutation) and 8 non-HCC liver tissues including live cirrhosis tissues and normal liver tissues. In immunohistochemistry IHC, TERT staining was observed both in the nucleus and cytoplasm although nuclear staining was more intense (Figure 3). The intensity of TERT expression in the nucleus and cytoplasm did not differ by promoter mutation status. However, the intensity of TERT staining was higher in HCC tissues compared to non-tumor liver samples (*p* = 0.001).

### *TERT* promoter hotspot mutations and TERT protein expression in the pre-neoplastic nodules

Dysplastic nodules may coexist with hepatocarcinoma tissues. We dissected preneoplastic lesions adjacent to the main HCC tissues from 7 cases (Supplementary Figure S3). In four of the 7 cases (57%), the C228T *TERT* mutation was found in both the paired preneoplastic lesions and the tumor tissue.

## DISCUSSION

*TERT* promoter mutations are one of the most frequent somatic alterations in a variety of human cancers [19, 30]. In the present study, we screened for the presence of telomerase promoter mutations in liver tumor tissues from 276 HBV-associated HCC patients and investigated the clinical relevance of this gain-of-function somatic mutation. We found that 31% of HCC tissue harbored *TERT* promoter mutations and that the *TERT* promoter mutations were more often detected in those with low AFP level. HCC tissues also had higher TERT protein expression than either cirrhotic or normal liver tissues in a small sub-dataset. On the other hand, we found no correlation between *TERT* promoter mutation status and clinical stage, tumor differentiation grades or cirrhosis, consistent with other studies [21, 22, 25].

*TERT* mutations occurred at an early stage of tumorigenesis as they were observed in 30% of stage I HCCs. *TERT* promoter mutations were previously found in isolated cirrhotic premalignant nodules (6-19%) [20, 21]. Unexpectedly, *TERT* mutations were observed in our small subset of 4 out of 7 (57%) of preneoplastic lesions adjacent to HCC nodules with the *TERT* mutations. The mutant T allele had generally lower peaks in the sequence graph in the precursor compared to cancer tissues, possibly reflecting a mixture of tumor and non-tumor cells (Supplementary Figure S3). It may be indicative of in-situ tumorigenesis in preneoplastic lesions but is also possible that cancer cells invaded into the adjacent dysplastic lesions. This finding and its implication requires further investigation.

The frequency of *TERT* promoter mutational events varies markedly in different global geographical regions. In our cohort comprising Han Chinese patients with HBV-associated HCC, 31% carried *TERT* mutations. This rate is comparable with those of HBV infected patients from other studies in East Asians (20.7% - 38.8%) [20, 22, 25], but is much lower compared to other continental groups: Europeans (47-59%) [20, 23], Americans (44%) [11], and Africans (53%) [24]. These data indicate that *TERT* promoter mutations are less frequent among Asians, most of whom have HBV-associated HCC. HBV integration in the *TERT* locus inducing transcriptional activation may partially account for the lower rate of *TERT* promoter mutations in HBV-related HCC compared to HCV or alcohol- related HCC [25, 31].

Serum AFP is a key tumor marker of HCC. Current guidelines are to suspect HCC in cirrhosis patients whose AFP level ≥400 μg/L for at least one month or ≥200 μg/L for at least two months [26, 27]. Our study indicated that *TERT* promoter mutations are more frequent in tumors from patients with lower levels of AFP, in agreement with the Nault et al study [20]. In this study, 51% (127/249) of HCC patients had false-negative AFP (< 20 μg/L), among whom 34.7% (n = 44) carried *TERT* mutations. Detection of cancer-specific *TERT* mutations in patients with low serum AFP may thus complement the AFP assay which has insufficient sensitivity and specificity for early diagnosis of HCC [4, 26, 27].

Nuclear detection of TERT protein is a strong indication of telomerase activation and ongoing cell proliferation [32, 33]. We observed higher nuclear expression of TERT protein in HCC tumors compared to adjacent or control non-tumor liver tissues; however, we observed no differences in *TERT* expression between tumor tissues with or without the *TERT* promoter mutations, consistent with previous mRNA studies [20, 22]. High TERT protein expression or telomerase reactivation is likely a fundamental event in HCC.

In addition, we observed that *TERT* promoter mutations in HCC were associated with older patient age, consistent with other studies in HCC [20, 22, 25] and in in several other cancers [34–37]. The age effect may indicate a higher probability of *TERT* mutation with advancing age.

We noted for the first time that *TERT* promoter mutations are rarely seen in the patients with HCC family history. It is possible that heritable germline genetic variation and non-*TERT* somatic gene mutations drive hepatocarcinogenesis in this subset. This relationship warrants further attention in future studies.

The small sample size used in the expression study limits the power to detect subtle differences among *TERT* genotype groups and we did not test for HBV integration into the *TERT* locus. HBV integration into the *TERT* locus was found in 50% of Japanese HBV-associated HCCs without *TERT* mutations [25]; HBV integration may activate TERT in those without promoter mutations [31]. Future studies are needed to clarify the role of HBV integration on the observed associations. Finally, the invasive nature of liver biopsies for detection of *TERT* mutation and TERT protein expression limits its clinical utility. The development of “liquid biopsies” should be explored in future studies.

In conclusion, *TERT* promoter mutations in HCC are common somatic mutations in Han Chinese infected with HBV, and are negatively correlated with family history and AFP serum level. *TERT* promoter mutations are not associated with TERT protein levels or HCC stages and grades. Detection of *TERT* promoter mutations in those with low levels of AFP may aid in the differential diagnosis of HCC with atypical presentation. Additional studies are required to determine if *TERT* promoter mutated-HCCs represent a subset of patients amenable to anti-TERT therapies.

## MATERIALS AND METHODS

### Patients and tumor tissue specimens

The study was conducted according to the principles in the Declaration of Helsinki and the national laws and regulations. The Ethics Committee of Wenzhou Medical University approved this retrospective study using archived pathological specimens and the de-identified health information (IRB00009844). The de-identified data were analyzed anonymously. An IRB exemption was obtained from the National Institutes of Health Office of Human Subjects Research (OHSRP Review #12836).

The tissue specimens were formalin-fixed and paraffin-embedded (FFPE) blocks that were archived at the Department of Pathology, from a consecutive series of 276 patients who underwent tumor resection at the First Affiliated Hospital of Wenzhou Medical University, Wenzhou, Zhejiang, China, during January 2010 and January 2014. Only specimens with pathologic diagnosis of hepatocellular carcinoma and of sufficient tissue specimens were used. The adjacent non-tumor preneoplastic lesions were separated on hematoxylin-eosin (HE) stained slides by microscopic-guided dissection, with aid of laser capture microdissection (ArcturusXT, Applied Biosystems) [38].

Tumor grading and staging were performed according to criteria of World Health Organization [39] and the Tumor Lymph Node Metastasis (TNM) classification of the international Union Against Cancer [40], which is based on tumor number, size, vascular invasion, lymphatic involvement and metastasis.

Of 276 HCC patients sequenced, 86% were male (n = 235) and 94% were HBV infected (based on HBV serological markers, among which 78% was HBsAg positive). Patients had a median age of 58 years (mean±SD, 56.5±11.2) ranging from 23 to 88 years old. Of 20 non-HCC controls, 16 had decompensated cirrhosis and 4 had hepatolithiasis. The study work flow is presented in Supplementary Figure S1.

AFP level in serum was measured using the Beckman Coulter Access alpha-fetoprotein (AFP) immunoassay kit on a Beckman Coulter UniCel DxI 800 instrument. The assay is a 2-site immunoenzymatic sandwich assay for the quantitative determination of AFP with a measuring range 0.50-51,000 ng/ml.

### DNA extraction

Genomic DNA from FFPE tissues was isolated from tissue sections with the phenol extract method [41]. Briefly, paraffin-embedded sample were subjected to xylene treatment to dissolves the paraffin, and then rehydrated. Digestion with Lysis buffer containing dodecyl sulfate (SDS) and proteins K was carried out at 56°C overnight, followed by phenol-chloroform extraction, isopropanol precipitation and re-suspension in H_2_O. Amount and quality of DNA were measured by a NanoDrop 2000 (Thermo Fisher Scientific).

### Sequencing of the *TERT* promoter region

The promoter region of *TERT* covering the two hotspot mutations -124C>T and -146C>T was amplified by polymerase chain reaction (PCR) as previously described [19]. The 163bp fragment of the TERT promoter region spanning the two hotspot mutations (−124, -146 sites) was amplified by KOD FXDNA polymerase (KFX-101, Toyobo Life Science) and performed with the following primer pairs: 5′-CAGCGCTGCCTGAAACTC-3′(sense) and 5′-GTCCTGCCCCTTCACCTT-3′(anti-sense). PCR products were visualized in 2% agarose gels and verified to have the expected size of 163 bp, which contained the site of C288T and C250T mutations. The direct sequencing was done on the 3730XL DNA Analyzer with BigDye Terminator V 3.1 (Applied Biosystems). Sequencing was carried out by BGI-Shanghai and Sunny Biotech Co., Ltd. (Shanghai, China). The detected mutations were validated in both strands.

### Immunohistochemistry (IHC) for TERT protein expression

FFPE slides of 3 μm sections were used to detect the TERT protein expression by IHC. Human TERT (hTERT) reactivity was detected by use of a specific mouse monoclonal [2C4] antibody to hTERT (ab5181, Abcam) with immunoperoxidase staining. Briefly, deparaffinized and rehydrated sections were exposed to 3% H_2_O_2_ solution for 10 min, followed by antigen retrieval in the citrate buffer. Incubation with anti-hTERT at a 1:100 dilution was carried out at 4°C overnight, followed by incubation with horseradish peroxidase-conjugated polymer anti-mouse secondary antibody for 20 mins at 37°C. 3,3′-Diaminobenzidine (DAB) (ZSGB-Bio) was applied for visualization. IHC score was evaluated by experienced pathologists. HCC slides without treatment of anti-hTERT were served as negative controls and tonsil slides were served as positive controls. The degree of positive staining for TERT was defined on the intensity of the staining and the percentage of stained cells [42]. In particular, the intensity was defined as negative (0), low (1), medium (2) and high (3), meanwhile the percentage of stained cells were defined as none (0), 1–10% (1), 10–50% (2) 50-80% (3) and >80% (4). We evaluated semi-quantitatively using the product of intensity and percentage of stained as following scale: negative (0-1); moderate (2-6); strong (8-12).

### Statistical analysis

We performed statistical analyses using SPSS software version 17.0 (SPSS Inc.). Student's t test was used to test the differences of continuous variables between groups with and without the TERT promoter mutations. Fisher's exact or χ^2^ test were used to examine associations between categorical variables. The results are expressed as a percentage or mean ± SD. A nonparametric analysis of Mann-Whitney U-test was used to test the immunostaining raw scores between the two groups because the analytical IHC scores were not normally distributed. All tests were two-tailed and results were considered statistically significant at *p*<0.05.

## SUPPLEMENTARY FIGURES AND TABLE


