# Supplementary material for: Telomerase reverse transcriptase promoter mutations in hepatitis B virus-associated hepatocellular carcinoma
Source: Oncotarget. 2016 Apr 1;7(19):27838–47. doi: 10.18632/oncotarget.8539 (PMC5053691; doi:10.18632/oncotarget.8539)
Supplement: Supplementary file 1 [file oncotarget-07-27838-s001.pdf]

Telomerase reverse transcriptase promoter mutations in hepatitis B virus-associated hepatocellular carcinoma

SUPPLEMENTARY FIGURES AND TABLE

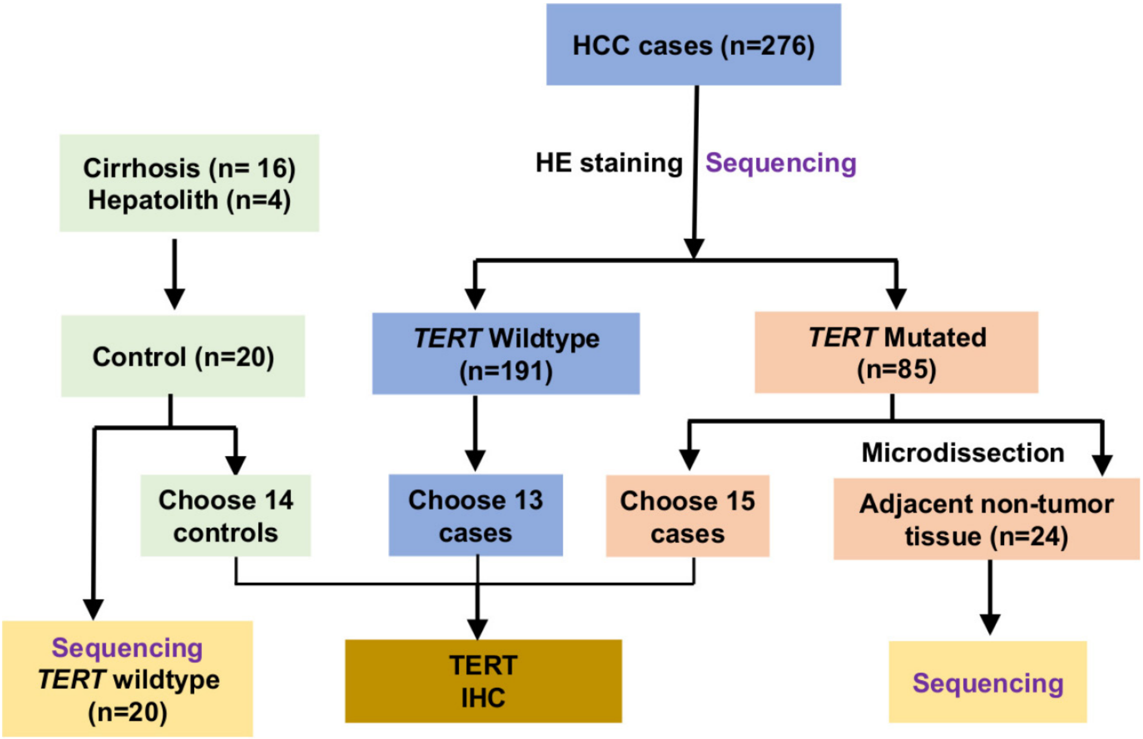

Supplementary Figure S1: Study design and work flow diagram for *TERT* promoter mutations in HCC.

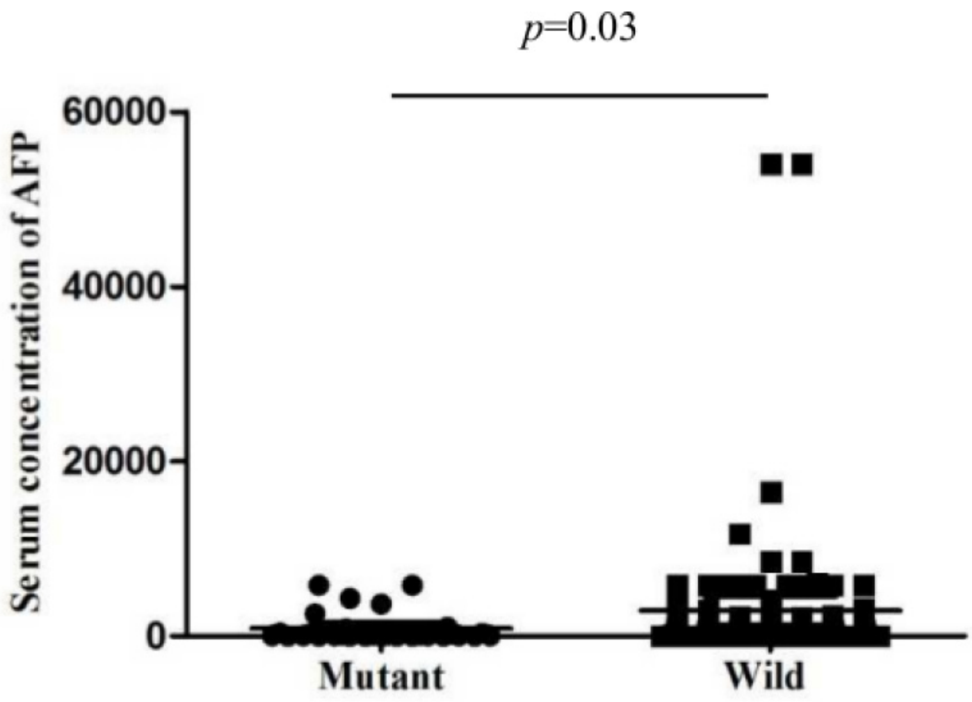

Supplementary Figure S2: The scatter plot of the concentration of AFP in different groups (*p* = 0.03) (include all data points) (Mean with 95%CI).

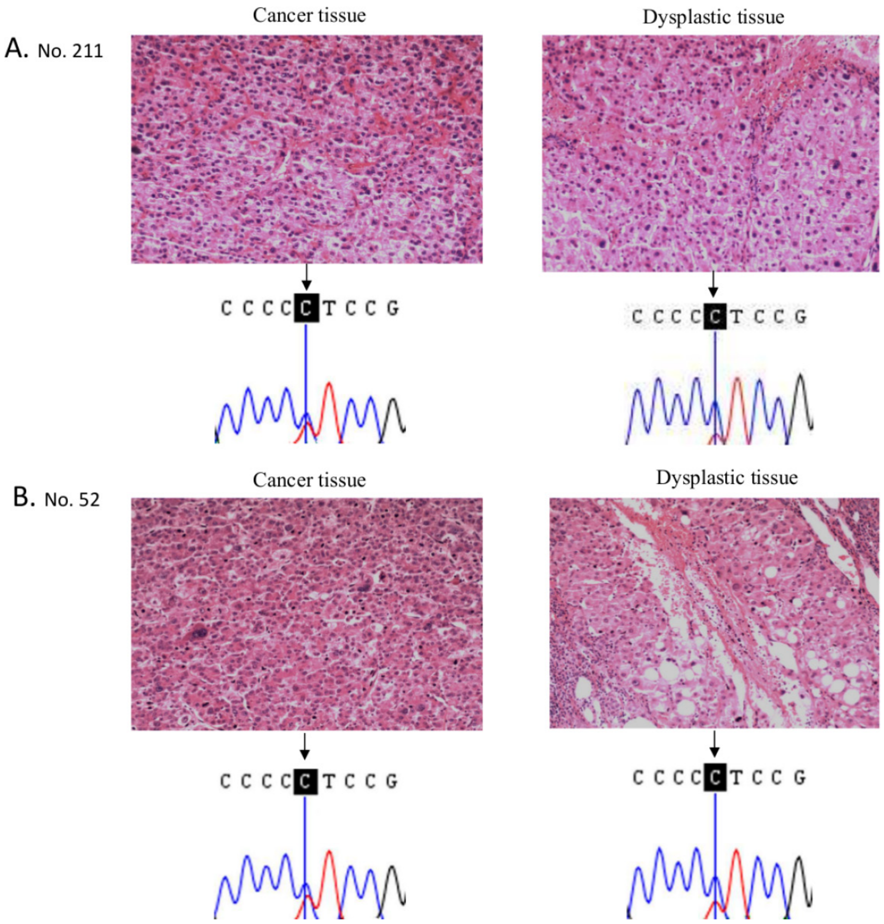

Supplementary Figure S3: *TERT* promoter mutations in HCC cancer tissue and dysplastic lesion from two representative cases (A and B).

Supplementary Table S1: Liver function tests of the HCC patients according to *TERT* promoter mutation status

Variable	*TERT* mutated Abnormal/total (%) n=85	*TERT* non-mutated Abnormal/total (%) n=191	OR	95%CI	*p* value*
TBIL	8/78 (10.3)	25/185 (13.5)	0.73	0.31-1.70	0.47
DBIL	18/79 (22.8)	39/180 (21.7)	1.07	0.57-2.01	0.84
IBIL	3/84 (3.6)	12/191 (6.3)	0.55	0.15-2.01	0.53
TP	14/85 (16.5)	39/191 (20.4)	0.77	0.39-1.51	0.44
ALB	13/85 (15.3)	29/191 (15.2)	1.01	0.50-2.05	0.98
ALT	12/85 (14.1)	42/191 (22.0)	0.58	0.29-1.17	0.13
AST	12/85 (14.1)	39/190 (20.5)	0.63	0.32-1.29	0.21
ALP	40/84 (47.6)	88/186 (47.3)	1.01	0.60-1.70	0.96
γ-GT	65/84 (77.4)	142/186 (76.3)	1.06	0.57-1.96	0.85

* χ^2 test.
